# The 2022 Indonesia Integrated Management of Childhood Illness (IMCI): Advantages of the Chart Booklet updates during the COVID-19 pandemic

**DOI:** 10.7189/jogh.13.03024

**Published:** 2023-06-16

**Authors:** Ida Safitri Laksono, Asal Wahyuni Erlin Mulyadi, Eggi Arguni, Fitri Haryanti, Suci Ardini Widyaningsih, Nurulita Ainun Alma, Nisa Rastiwi

**Affiliations:** 1 Department of Child Health, Faculty of Medicine, Public Health and Nursing, Universitas Gadjah Mada, Yogyakarta, Indonesia; 2 Center for Child Health-PRO, Faculty of Medicine, Public Health and Nursing, Universitas Gadjah Mada, Yogyakarta, Indonesia; 3 Department of Public Administration, Faculty of Social and Political Sciences Universitas Sebelas Maret Surakarta, Central Java, Indonesia; 4 Department of Pediatric and Maternity Nursing, Faculty of Medicine, Public Health and Nursing, Universitas Gadjah Mada, Yogyakarta, Indonesia

**Figure:**
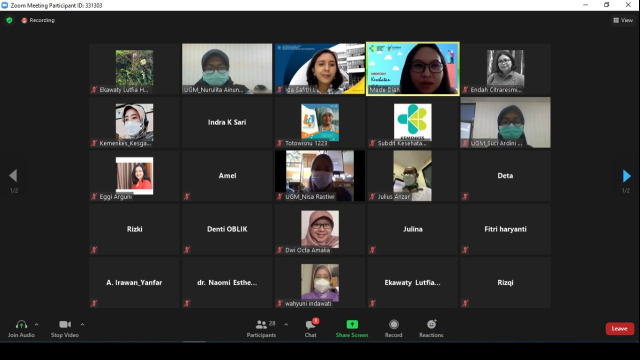
Photo: One of the desk review activities via virtual meeting with Indonesia Ministry of Health, WHO Indonesia, and the Indonesian Pediatric Society (IDAI). Source: Center for Child Health-PRO documentation, used with permission.

The Integrated Management of Childhood Illness (IMCI) is a guideline to decrease mortality, morbidity, and disability in children under five. The IMCI guides doctors, nurses, midwives, and health cadres to manage illness-causing mortality, for instance, pneumonia, diarrhoea, malaria, dengue infection, measles, tuberculosis, Human Immunodeficiency Virus (HIV), anaemia, and malnutrition [1]. The IMCI Chart Booklet is a tool to implement the IMCI guideline.

Management of sick children using the IMCI approach is cost-effective and widely contributes to decreasing neonatal, infant, and child mortality [2]. The mortality rate of children under five in Indonesia in 1997 was 58 per 1000 live births. It declined to 32 per 1000 live births in 2017 after IMCI was implemented in Indonesia [3]. However, according to the Sustainable Development Goals targets, the under-five mortality rate should be reduced to 25 per 1000 live births by 2030 [4]. Deaths due to preventable diseases in newborns and children should be eliminated by maintaining health and well-being among children [5]. The 2015 IMCI is the latest version implemented in Indonesia that should be updated. According to Technical Updates of the Guidelines on the IMCI,‎ World Health Organization (WHO) recommends to updating IMCI based on the recent guidelines and research about clinical management [6]. In addition, updates should be considered when the epidemiological profile at regional and national levels has changed [7].

In 2020-2022, during the COVID-19 pandemic, we conducted a desk review to update the last version of the 2015 IMCI Chart Booklet. This article aimed to elucidate the process and the benefits of updating the 2022 Indonesia IMCI Chart Booklet.

## DESK REVIEW ON THE IMCI CHART BOOKLET DURING THE COVID-19 PANDEMIC

The IMCI was developed by the WHO in 1994 [8]. This guideline was introduced in Indonesia in 1997 [1]. The data from Health Facility Research in 2011 mentioned that approximately 80% of primary health care in Indonesia had implemented the IMCI [9]. Previously, the IMCI Chart Booklet was revised in 2003, 2008, and 2015. Before the COVID-19 pandemic, IMCI revisions were usually through serial face-to-face meetings held by the Ministry of Health (MoH) in Jakarta, the capital city of Indonesia. The sessions invited experts and IMCI facilitators from many provinces.

The fourth revision of the Indonesia IMCI Chart Booklet was conducted according to national conditions, policies, and guidelines. In the first activity, the Directorate of Family Health MoH initiated and proposed parts of the IMCI Chart Booklet that should be revised from cross programs and cross sectors. The Center for Child Health-PRO, Faculty of Medicine, Public Health, and Nursing, Universitas Gadjah Mada was assigned to lead the revision process. The revision team implemented a desk review method to collect, organize and synthesize resources from the latest evidence at global and national levels. The revision process consists of five main activities: 1) coordination with the Indonesian MoH and WHO Indonesia, 2) desk reviews with external stakeholders, 3) desk reviews with internal stakeholders, 4) dissemination and feedback, and 5) final review.

The COVID-19 pandemic prompted the revision team to perform the desk review via online and offline discussions. Virtual meetings were chosen when we discussed with MoH, professional organizations, and stakeholders due to several reasons. They were located in different towns and there were travel limitations during the pandemic, such as requirement to perform COVID-19 tests. Thus, it is costly to arrange direct meeting with stakeholders from different towns. On top of that, the Government of Indonesia restricted the number of people who gathered in one place [10]. Online discussions were conducted through Zoom Meeting because of several considerations. First and foremost, MoH provided the Zoom premium account to arrange virtual meetings. Based on a national survey about remote learning in nursing students, Zoom Meeting was the most popular platform for synchronous online meeting tools [11]. Second, Zoom Meeting is easily accessed in computers, laptops, and smartphones. Third, it could save the recording of the meetings in the form of video and voice. Therefore, the revision team could review the recording as the materials to draft the IMCI Chart Booklet. Meanwhile, direct meetings were conducted for discussions with paediatric consultants from the Faculty of Medicine, Public Health and Nursing, Universitas Gadjah Mada, Yogyakarta because they reside in the same town with the revision team.

**Figure 1:**
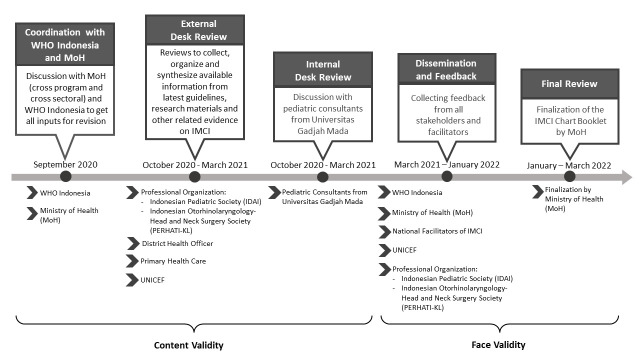
The desk review activities of the 2022 Indonesia Integrated Management of Childhood Illness (IMCI) Chart Booklet.

The desk review was conducted from September 2020 until March 2022 (**Figure 1**). The process started from a discussion with MoH in September 2020. Afterwards, the revision team arranged meetings with external stakeholders from professional organizations. The revision process was followed by discussions with internal stakeholders from the Faculty of Medicine, Public Health and Nursing, Universitas Gadjah Mada. Then, a focused group discussion (FGD) with district health officers and primary health care representatives was performed. The revision process continued to the dissemination process. Then, the revision team obtained feedback from all the stakeholders before the updated IMCI was finalized by the MoH in Jakarta.

## UPDATES OF THE IMCI CHART BOOKLET

Through the desk review method, the content of the 2022 IMCI Chart Booklet is more updated and comprehensive. There are several changes compared to the 2015 IMCI Chart Booklet edition, for instance, the layout, new sick child classification, and disease management approaches that are adapted from the latest guidelines.

**Figure 2:**
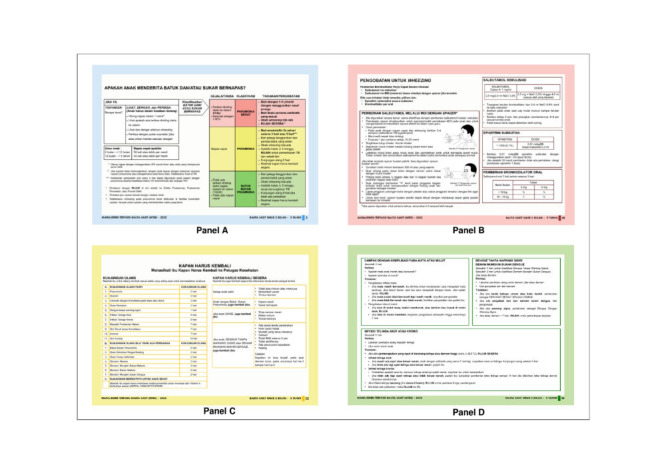
New colour system to differentiate the chapter on Integrated Management of Childhood Illness (IMCI) Chart Booklet. **Panel A.** Blue for sick child classification. **Panel B.** Pink for treatment. **Panel C.** Yellow for counselling. **Panel D.** Green for follow up.

The IMCI chapters consist of sick child classifications, treatments, counselling, and follow-up care. Each chapter has a different frame colour to simplify and increase the user's enthusiasm. The colour for sick child classification is blue, treatment is pink, counselling is yellow, and follow-up is green (**Figure 2**). These colours will ease the user to find the appropriate chapter, which are not found in the previous edition of the Indonesia IMCI Chart Booklet.

Moreover, the classification is updated based on the latest guideline. Updates are needed to accommodate changes in disease epidemiology. For example, dengue fever cases increased significantly in 2019 at local and global levels [12]. In addition, Indonesia has new National Guidelines for Medical Services on Case Management of Dengue in Children and Adolescents, which are in line with the WHO Dengue Guideline 2009. The 2022 Indonesia IMCI Chart Booklet also emphasizes dengue warning signs. Therefore, doctors, nurses, and midwives are more competent in classifying dengue.

The newest 2022, version of IMCI Chart Booklet is more comprehensive as it includes additional classifications of stunting and paediatric emergency. The first classification is stunting. Stunting is included in the section on Sustainable Development Goals, the 2025 Global Nutrition Targets, as adopted by the 65th World Health Assembly in 2012 [13]. In 2017, the National Strategy to Accelerate Stunting Prevention (StraNas Stunting) was launched to tackle stunting in Indonesia [14]. Previously, there is no classification about stunting in the IMCI Chart Booklet. By adding this classification, doctors, midwives, and nurses in frontline health facilities are encouraged to screen stunting. Thus, they can plan for further management of stunting. Comprehensive screening is needed from the most basic health care facility.

The second new classification is paediatric emergencies, adapted from the Pediatric Assessment Triangle (PAT). It is a simple instrument to assess children in emergency conditions rapidly. Three elements need to be observed, such as, appearance, work of breathing, and circulation to the skin [15]. Indonesia Ministry of Health in 2019 found about 21.3% of neonatal mortality was caused by respiratory and cardiovascular diseases [16]. WHO stated that children's mortality happened in the first 24 hours after being admitted at the hospital. It is influenced by many factors, for example the state of the patients on arrival, triage system and time response [17]. Therefore, the topic about paediatric emergency triage is needed to be added to the IMCI Chart Booklet.

## ADVANTAGES AND LESSONS LEARNT

Due to the social distancing practice, virtual conferences have become the norm for meetings during the COVID-19 pandemic. These virtual meetings bring merits [18]. The first benefit of the online IMCI revision activities is the process involved many stakeholders, for instance, governments, professional organizations, experts, district health officers, and primary health care representatives. In the beginning, the revision team arranged meetings with the Directorate of Family Health MoH.

The discussion also involved paediatric consultants at the Faculty of Medicine, Public Health and Nursing, Universitas Gadjah Mada, as the internal stakeholders, such as paediatric respirology, neurology, and neonatology. In addition, there were several discussions with external stakeholders from professional organizations, for instance, the Indonesian Pediatric Society (IDAI), which consists of several working groups and task forces, ie, neonatology, infection and tropical diseases, nutrition and metabolic diseases, gastro-hepatology, respirology, hemato-oncology, paediatric emergency and intensive care, paediatric pharmacy task force, HIV, and immunization. The other professional organization includes the Indonesian Otorhinolaryngology-Head and Neck Surgery Society (PERHATI-KL).

Moreover, the virtual FGD was conducted to obtain perspectives on the IMCI implementation from external key stakeholders, such as district health officers and primary health care representatives. Those stakeholders were from Yogyakarta Province as the sample region of IMCI implementation. The results of the FGD explained that the majority of the health care workers have not obtained training and the IMCI implementation should be improved [19].

National facilitators of IMCI are also involved in the review process. They gave insights about training delivery of IMCI, for instance the potential difficulty of IMCI training using the new IMCI Chart Booklet. Therefore, the structure and content of the new IMCI Chart Booklet will be more easily understood by health care workers in frontline facilities in Indonesia.

All stakeholders contributed to the dissemination and feedback before the final review of the new IMCI Chart Booklet. The first session of the dissemination presented the IMCI algorithm for children between two months to five years. Meanwhile, the second session discussed the young infant algorithm.

In addition, the combination of virtual and direct meetings is less costly and more efficient in time. Previously, IMCI revisions were conducted through offline meetings which invited many experts and stakeholders. Since using online and offline methods, this desk review can decrease the budget for direct meetings, such as accommodations, transportation, refreshments, and materials. This advantage is supported by a study among Egyptian oncologists, which mentioned that virtual education and meeting activities during COVID-19 pandemic in the oncology field in Egypt could save on travel expenses [20].

Via virtual meetings, discussions were conducted frequently and intensely. The dissemination of the 2022 IMCI Chart Booklet also could be extensive because the MoH invited stakeholders of all provinces in Indonesia, including from remote areas. Thus, the discussions could enrich the content of the IMCI Chart Booklet.

Also, the revision period was in the right circumstance because several related guidelines from the Ministry of Health and IDAI were being revised, such as guidelines about stunting, dengue, immunization schedule, and paediatric emergency.

Furthermore, the combination of online and offline meetings could ensure the safety of the desk review. Since the revision was held during the peak of the COVID-19 pandemic, virtual meetings can prevent COVID-19 transmission because they can minimize direct contact between humans.

Even though the virtual meeting has many advantages, there were some obstacles. When the meeting was attended by many participants, we had difficulties in managing them to be equally participated in the discussion. In addition, some participants had unstable internet connection.

## CONCLUSION

The COVID-19 pandemic compelled the 2022 Indonesia IMCI revision team to conduct the desk review of the IMCI Chart Booklet through a combination of online and offline methods. There were benefits in several aspects, and this revision process was less costly, time-efficient, more updated, and comprehensive. Therefore, online meetings only or a combination of virtual and direct meetings (hybrid) could be a strategy to update guidelines in the future, not only during the COVID-19 pandemic.
